# Arrayed CRISPR reveals genetic regulators of tau aggregation, autophagy and mitochondria in Alzheimer’s disease model

**DOI:** 10.1038/s41598-021-82658-7

**Published:** 2021-02-03

**Authors:** Lishu Duan, Mufeng Hu, Joseph A. Tamm, Yelena Y. Grinberg, Fang Shen, Yating Chai, Hualin Xi, Lauren Gibilisco, Brinda Ravikumar, Vivek Gautam, Eric Karran, Matthew Townsend, Robert V. Talanian

**Affiliations:** 1 Cambridge Research Center, AbbVie, 200 Sidney Street, Cambridge, MA 02139 USA; 2 AbbVie Inc., 1 North Waukegan Rd., North Chicago, IL 60064 USA

**Keywords:** Cell biology, Computational biology and bioinformatics, Drug discovery, Genetics, Molecular biology, Neuroscience

## Abstract

Alzheimer’s disease (AD) is a common neurodegenerative disease with poor prognosis. New options for drug discovery targets are needed. We developed an imaging based arrayed CRISPR method to interrogate the human genome for modulation of in vitro correlates of AD features, and used this to assess 1525 human genes related to tau aggregation, autophagy and mitochondria. This work revealed (I) a network of tau aggregation modulators including the NF-κB pathway and inflammatory signaling, (II) a correlation between mitochondrial morphology, respiratory function and transcriptomics, (III) machine learning predicted novel roles of genes and pathways in autophagic processes and (IV) individual gene function inferences and interactions among biological processes via multi-feature clustering. These studies provide a platform to interrogate underexplored aspects of AD biology and offer several specific hypotheses for future drug discovery efforts.

## Introduction

Alzheimer’s disease (AD) is a prevalent, aggressive neurodegenerative disease for which effective treatments are lacking^1^. While autosomal dominant genetic variants have suggested key initiating factors^2^, those critical for pathology progression and ultimate dementia largely remain to be elucidated. We set out to reveal such mechanisms by screening for modulators of key pathological phenotypes of AD in an in vitro model.

Increasing evidence supports tau as a therapeutic target, as human data demonstrate that the extent of tau pathology correlates better than amyloid with AD disease progression, brain atrophy and cognitive decline^3,4^. Pathogenic variants of the MAPT gene, encoding tau protein, can cause a range of neurodegenerative diseases known as tauopathies. Tau-targeting strategies include small molecules and immunotherapies that modulate tau protein levels, post-translational modifications, and aggregation^5^. However, many pharmacological agents have been limited by toxicity or lack of efficacy^5^. Therapeutic antibodies targeting tau achieve limited brain exposure due to the blood–brain barrier (BBB) and do not access intracellular tau tangles.

Many biological processes are altered in AD^6^. These might be crucial mediators of Aβ and tau toxicity or may contribute to AD independently. Mitochondrial structural and functional differences in AD patients have led to the mitochondrial cascade hypothesis of AD^7^. Proteostatic mechanisms are also disrupted in AD, particularly autophagy, which mediates large molecule (e.g., tau aggregate) degradation by lysosomes^8^. Mounting evidence from genome-wide association studies (GWAS) suggests a key role for innate immunity in AD, particularly mediated via microglia^9,10^. These mechanisms, their interactions, and their potential for pharmacological modulation, are partially understood at best. A systematic experimental approach to identify genes that modulate protein aggregation, the associated proteopathic stress, as well as cellular responses to that stress before irreversible disease progression, may advance that understanding and lead to new AD drug targets^9^.

Functional genomics allows direct and unbiased interrogation of the human genome for new drug targets. RNA interference (RNAi) and open reading frame (ORF) methods have been applied at the whole genome scale. While hugely impactful, these methods need orthogonal approaches to get a more complete view of the potential target landscape due to limitations such as widespread off-targeting, short target inhibition duration, and supraphysiological concentration-elicited non-specific cellular effects^11,12^. Pooled CRISPR knockout (KO) screens have generally been more specific and consistent than RNAi screens^13,14^. Pooled CRISPR screens in neurodegenerative diseases face several challenges, including (i) inability to interrogate early disease-relevant processes prior to survival endpoints used in traditional drop-out pooled CRISPR screen methods, (ii) difficulties in capturing morphological features, cellular localization, temporal sequences, and intracellular events by positive enrichment pooled CRISPR screen methods, and (iii) missing non-cell autonomous effects and multimodal interactions. These can be overcome by the arrayed CRISPR format, in which individual genes are knocked out in individual wells, followed by high content imaging (HCI). Publications of large-scale arrayed CRISPR screens have been limited, possibly due to laborious processes, limited availability of high-performance libraries, and challenges in quality control to achieve high and consistent editing efficiency to minimize phenotypic heterogeneity.

We developed an automated, arrayed CRISPR high content imaging phenotypic screen workflow, and used it to evaluate in an in vitro cell model system tau aggregation, autophagy, lysosomes, mitochondria, Golgi and cell health in parallel. A pilot screen targeting 1525 genes confirmed and extended biology reported in the literature, and revealed novel biology in each cellular process. A network of genes involved in immune response modulated tau aggregation including the NF-κB pathway and LKB1 complex. We present evidence that mitochondrial morphology correlates well with respiratory function and gene expression profile. Further, we predicted and inferred gene functions as well as interaction of biological processes by applying bioinformatic tools. These methods and findings advance our understanding of tau pathobiology, providing novel target hypotheses and technical platforms to inform AD drug discovery efforts.

## Results

### Automated multi-modality arrayed CRISPR phenotypic screen in AD relevant cell model

**Figure 1:**
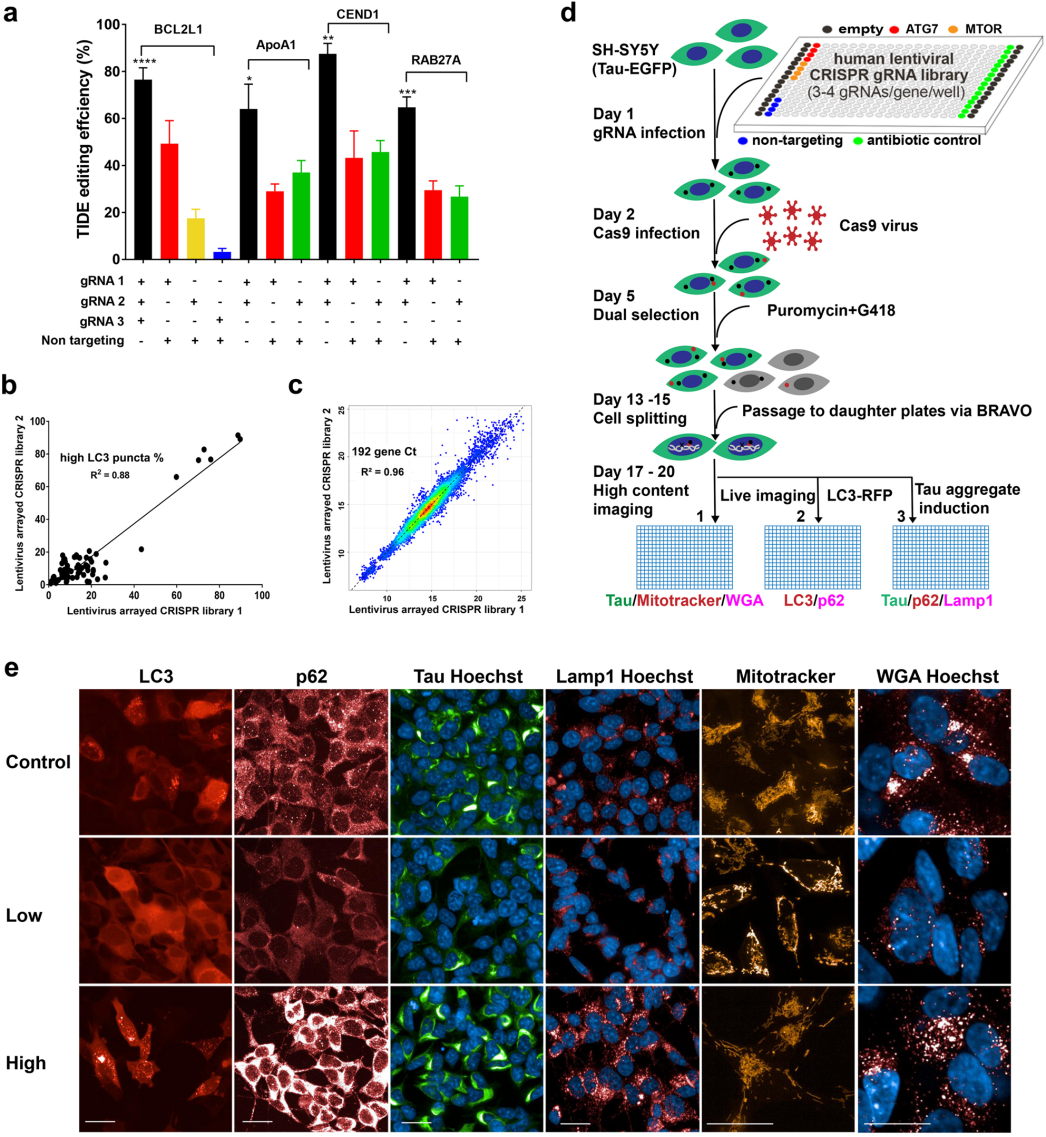
Multi-modal arrayed CRISPR and HCI phenotypic screen development. (**a**) Multiplexing lentiviral gRNAs significantly increased editing efficiency compared to single guides. Total lentivirus volume was fixed, n = 4, one-way ANOVA was applied to compare gRNAs performances in each gene group, *p-value < 0.05 (*p* = 0.016), **p-value < 0.01 (*p* = 0.004), ***p-value < 0.001, ****p-value < 0.0001. (**b**) Pilot CRISPR screen with two batches of lentiviral gRNA preparation reveals significantly correlated functional phenotype—percentage of cells high in LC3 puncta. R^2^ = 0.88, *p*-value < 0.0001. (**c**) qPCR analysis of 192 genes resulting from pilot CRISPR screen show highly correlated expression indicated by Ct values. R^2^ = 0.96, *p*-value < 0.0001. (**d**) Automated 384-well arrayed CRISPR and HCI screen workflow. (**e**) Representative confocal images of screen phenotypes, highlighting low and high signals in number of puncta, signal intensity, spectrum of morphology for each readout respectively, scale bar = 10 μm.

We developed an arrayed CRISPR phenotypic screening method using engineered SH-SY5Y neuroblastoma cells to interrogate the genetic component of established AD pathologies. Misfolded tau aggregation can be modelled in SH-SY5Y cells by overexpressing full length 2N4R tau containing the human tauopathy-associated P301L mutation and tagged with C-terminal EGFP to visualize tau aggregation in vitro. Upon treatment with synthetic tau fibrils, these cells form intracellular detergent-insoluble hyperphosphorylated tau aggregates^15,16^. Cas9 and gRNAs were delivered by lentivirus, and virus titer and timing were optimized for CRISPR editing efficiency and phenotypic translation (Supplement Fig. 1). Editing efficiency was further increased by multiplexing gRNAs. At a fixed total lentiviral input, multiplexed gRNAs consistently achieved 65% or higher editing while individual gRNAs varied from 3 to 45% (Fig. 1a). In a pilot screen of multiplexed gRNAs targeting 63 proteostasis genes, two independently prepared lentiviral gRNA libraries produced reproducible changes in the phenotypic endpoint (LC3 puncta) (R^2^ = 0.88, *p* < 0.0001) (Fig. 1b). A qPCR analysis demonstrated that the expression profile of 192 genes was highly correlated between lentiviral libraries (R^2^ = 0.96, *p* < 0.0001) (Fig. 1c). Thus, although lentivirus preparations may vary in titer, the phenotype produced by CRISPR editing can be highly consistent. This reproducibility could be due to high CRISPR editing efficiency as well as on-target specificity. These results indicate that large scale lentiviral arrayed CRISPR screen can be used for phenotypic screening.

RNA-seq analysis was used to compare the gene-specific molecular changes mediated by three different methods: CRISPR, siRNA and ORF, utilizing different constructs targeting the same genes. As expected, ORF overexpression led to significantly different gene expression profiles than CRISPR and siRNA, with thousands of gene changes (compared to hundreds), accounting for 24% of the variance by principal component analysis (PCA) (Supplemental Fig. 1). Hierarchical clustering of differentially expressed genes (DEG—log2 fold change) induced by CRISPR or siRNA revealed that while individual gRNAs targeting the same gene had similar mRNA profiles, siRNAs showed distinct expression profiles, in agreement with published results^17^ (Supplemental Fig. 1). Together, these data suggest that an optimized multiplexed gRNA arrayed CRISPR protocol produce consistent and gene-specific cellular and molecular profiles.

We scaled this up to evaluate for modulation of AD relevant phenotypes in vitro, 1525 select human genes that we categorically favor as “druggable”, particularly those annotated as kinases, phosphatases, and epigenetics-associated genes (Supplemental Fig. 2). 3–4 gRNAs were pooled per gene/well and on plate controls were included to monitor screen performance. Automation enabled high-throughput 384-well cell splitting so that all phenotypic readouts were derived from the same gene editing event (Fig. 1d).

**Figure 2:**
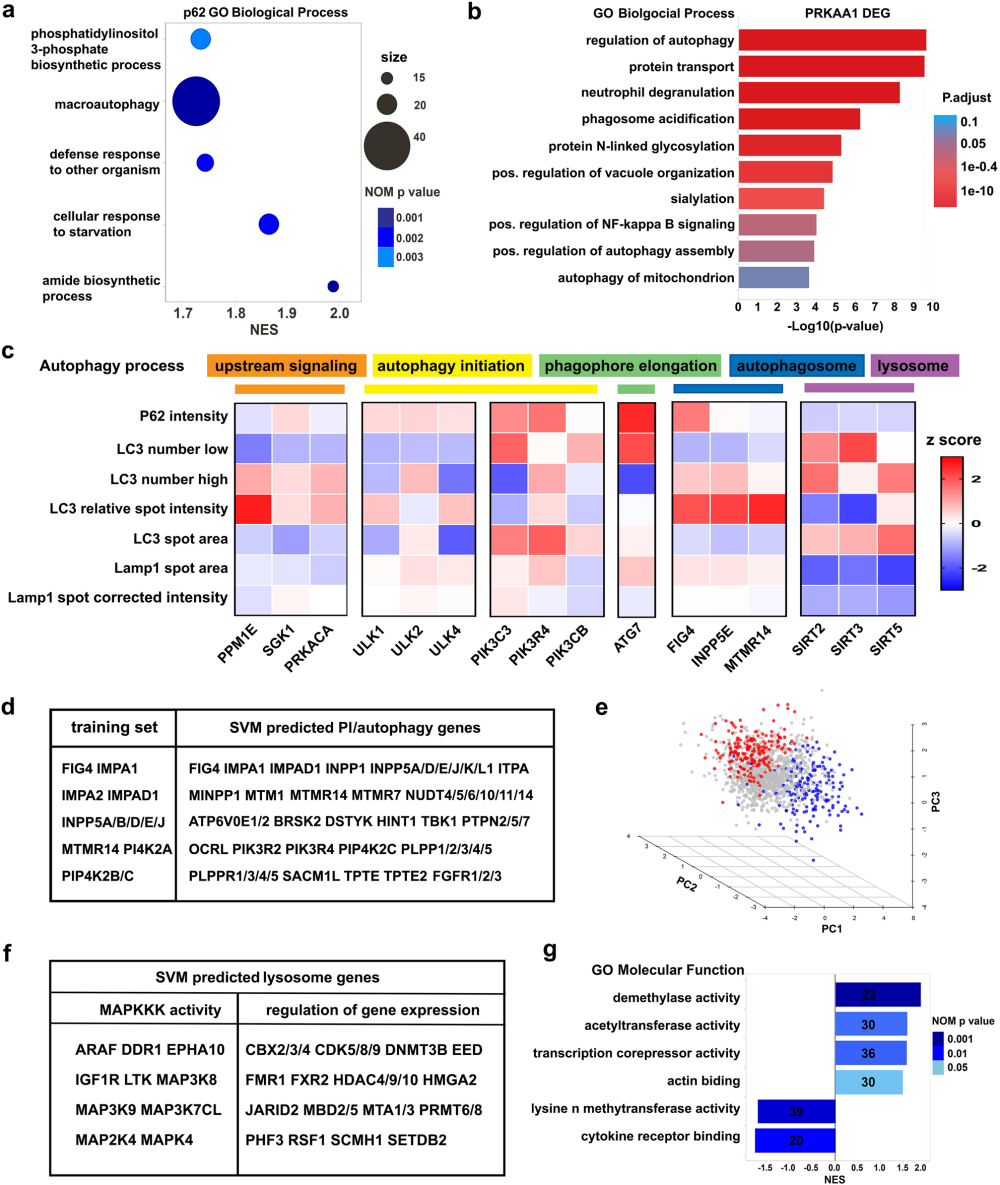
Basal autophagy and lysosome gene discovery via arrayed CRISPR and multi-parameter HCI. (**a**) GSEA analysis of p62 z score ranked gene list reveal enriched GO Biological Processes such as macroautophagy. (**b**) Enrichment analysis of differentially expressed genes upon *PRKAA1* CRISPR show perturbed biological processes consistent with its role in autophagy. (**c**) Panel of autophagy related HCI parameters capture distinct patterns for genes involved in different stages of autophagy. d and f, Support-vector machine (SVM) learning was applied to 7-parameter dataset to predict genes that act similarly to phosphoinositol (referred to as PI) related genes in autophagy processes (**d**) and sirtuin genes in lysosome homeostasis (**f**). (**e**) Illustration of SVM method, predicted genes highlighted in red and blue, background is in grey. (**g**) Epigenetic regulators impact lysosome bi-directionally, supported by enriched pathways from GSEA analysis of LAMP1 z score ranked gene list.

The arrayed CRISPR screen performed robustly. Sampling of 45 gRNAs targeting 15 genes revealed high editing efficiency (> 50% per gene) and plate controls performed as expected (Supplemental Fig. 2). Representative images of screening phenotypes are shown (Fig. 1e). To reveal biological insights from the dataset, i.e., gene function, pathway crosstalk, and interactions of biological processes, we developed a data analysis and bioinformatic pipeline (Supplemental Fig. 2).

### Arrayed CRISPR screen and machine learning uncover novel genes and pathways that impact basal autophagy and lysosome homeostasis

A key mechanism for intracellular macromolecule degradation is autophagy. Cytoplasmic autophagy receptors such as p62/SQSTM1 link ubiquitinated macromolecules to nascent LC3-positive autophagosomes. As autophagosomes mature they fuse with lysosomes leading to the degradation of their contents^8^. To interrogate broadly the genetic underpinnings of basal autophagy and lysosome homeostasis, we monitored autophagy by p62 and LC3-RFP, and lysosomes by LAMP1 staining, while selectively disrupting 1525 genes (Fig. 1e). The data was quantified and normalized by calculating plate-based z-scores. Gene set enrichment analysis (GSEA) of the p62 intensity z-score ranked gene list confirmed a significant enrichment of autophagy-related genes including macroautophagy, phosphatidylinositol-3-phosphate biosynthetic process, defense response, and cellular response to starvation (Fig. 2a). This demonstrates that the arrayed CRISPR screen can robustly identify cellular pathways despite screening only selected subsets of genes.

Transcriptional profiling was performed to assess alignment of molecular profiles with cellular findings. Knockout of *PRKAA1*, encoding the α subunit of AMPK, elicited a transcriptional response highly enriched for autophagy and lysosomal processes, consistent with the role of AMPK in autophagy and lysosome regulation^18^ (Fig. 2b). We also observed enrichment of novel gene sets involved in protein modification, such as glycosylation and sialyation, and NF-κB signaling (Fig. 2b). This suggests that AMPK has a role as a master homeostasis and metabolic regulator, which merits further investigation. More broadly, the results show the feasibility of large-scale CRISPR perturbation followed by transcriptional profiling to decipher gene function.

In the literature, assessing the state of cellular autophagy typically includes quantification of LC3 and p62 positive autophagosome numbers^19^. Expanding the variety of features measured by HCI to include autophagosome size, intensity, percentage of cells exhibiting high or low LC3 puncta, as well as a LAMP1 marker, can further differentiate subtle phenotypes. To test this, seven HCI parameters were used to evaluate the effects of a set of autophagy-modulating tool compounds (Torin1, rapamycin, chloroquine and bafilomycin A1) with distinct mechanisms of action. These newly established detection parameters clearly differentiated the phenotypes induced by the four test compounds in SH-SY5Y cells (Supplemental Fig. 3).

**Figure 3:**
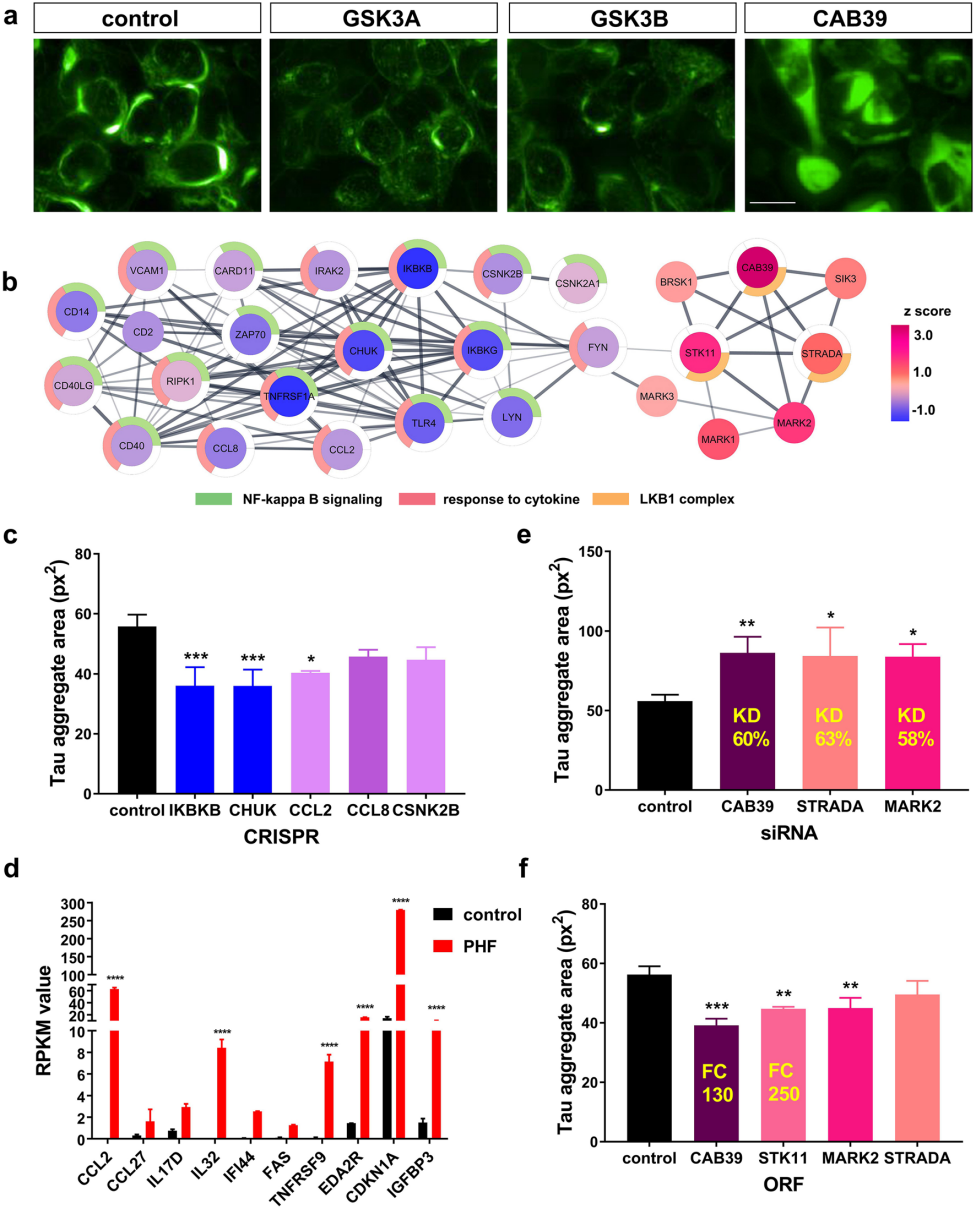
Discovery of interconnected network of inflammatory NF-κB pathway and LKB1 complex that modulate tau aggregation. (**a**) Tau-EGFP aggregate morphology after methanol fixation to remove soluble tau. *GSK3A/B* knockout decreases tau aggregate area while *CAB39* knockout increases it. Scale bar = 10 μm. (**b**) Cytoscape construction of STRING network of gene sets that modulate tau aggregation, colored by aggregation area z score. Outer ring highlight network enriched pathway. STRING interaction was limited to experimental evidence and database. (**c**) Representative inflammatory and NF-κB pathway gene hits are confirmed in secondary CRISPR experiments n = 4, one-way ANOVA, F (5,12) = 9.503, *p* = 0.0007; Sidak’s multiple comparisons, *p = 0.014. (**d**) Tau aggregate bearing cells upregulate expression of inflammatory genes detected by RNA-seq, n = 2, Two-way ANOVA with Sidak’s multiple comparisons, F (9, 20) = 9371, *p* < 0.0001. (**e**) siRNA knockdown of LKB1 complex related genes repeat tau aggregation increase phenotype by CRISPR method, knockdown efficiency (KD) determined by QPCR is highlighted in yellow, n = 4, one-way ANOVA, F (3, 12) = 6.687, *p* = 0.0066; Sidak’s multiple comparisons, *CAB39* *p* = 0.007, *STRADA* *p* = 0.011, *MARK2* *p* = 0.013. (**f**) Over-expression of LKB1 complex related genes decrease Tau aggregation, further confirming primary screen finding. Over expression fold change (FC) by qPCR is indicated in yellow, n = 3, one-way ANOVA, F (4, 10) = 13.11, *p* = 0.0005; Sidak’s multiple comparisons, *STK11* *p* = 0.004, *MARK2* *p* = 0.004. All error bars indicate standard deviation, *p-value < 0.05, **p-value < 0.01, ***p-value < 0.001, ****p-value < 0.0001.

**Figure 4:**
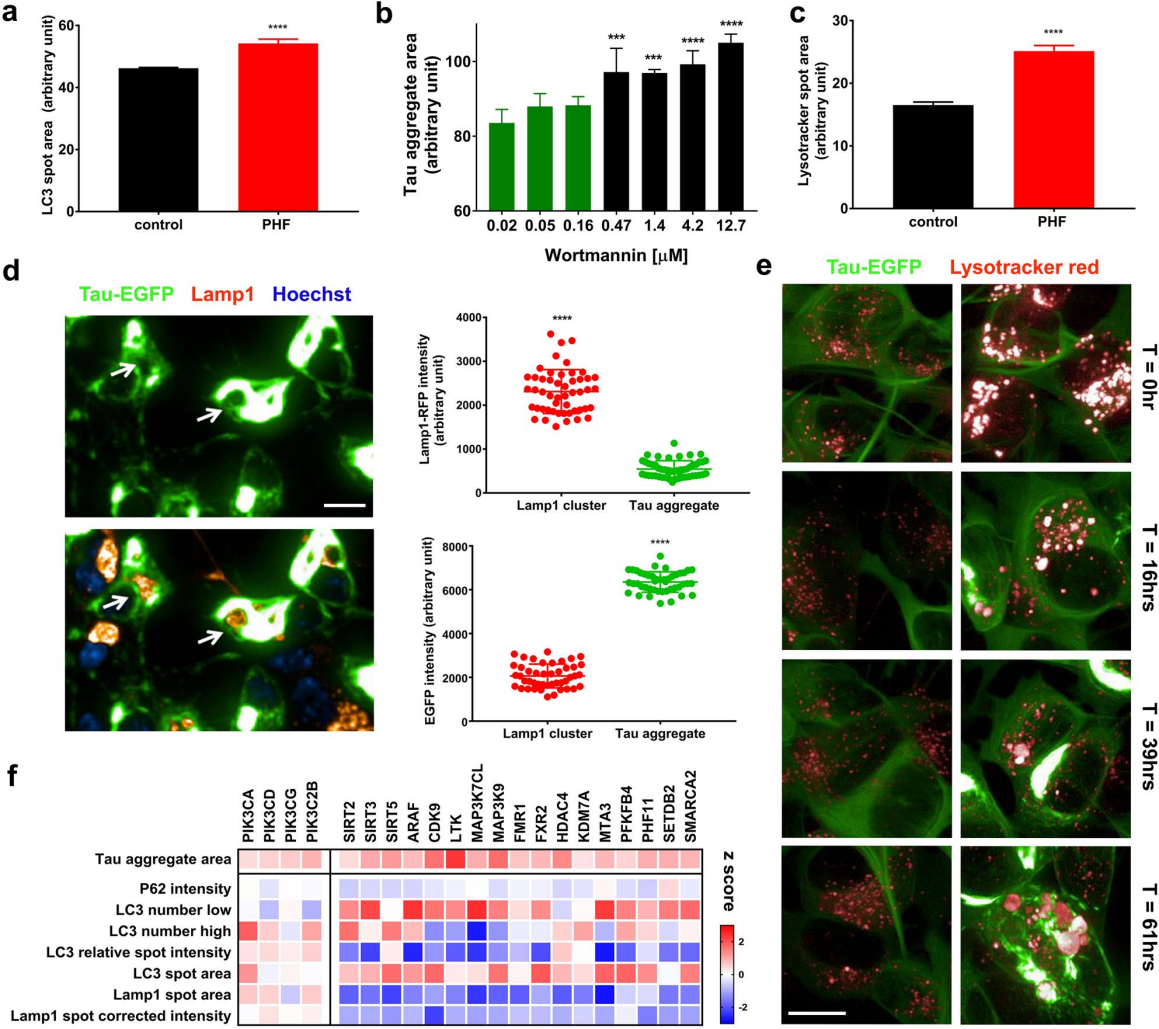
Interaction of tau aggregation with autophagosome and lysosome dynamics. (**a**) PHF-treated cells display significantly enlarged LC3 positive autophagosome, n = 4, two-tailed unpaired t-test. (**b**) pan-PI3K autophagy inhibitor wortmannin concentration dependently increases tau aggregate spot area, n = 4, One-way ANOVA with Tukey’s multiple comparisons, F (6, 20) = 19.74, *p* < 0.0001. (**c**) PHF-treated cells significantly increase lysosome size marked by lysotracker red, n = 5, two-tailed unpaired t-test. (**d**) LAMP1-positive lysosome clusters and tau-EGFP aggregates are mutually exclusive, two-tailed unpaired t-test. (**e**) Live imaging of lysosome coalescence via lysotracker red during tau aggregation process, scale bar = 10 μM. (**f**) Tau aggregation, autophagy and lysosome profiles of examples genes similar to wortmannin and *SIRT2*. Error bars indicate standard deviation. ***p-value < 0.001, ****p-value < 0.0001.

Applying these parameters to CRISPR edited cells revealed signaling pathways involved in different autophagy stages. These could be binned into five subpopulations: upstream signaling mediators; autophagy initiation; phagophore elongation; autophagosome maturation and autolysosome/lysosome function (Fig. 2c). For example, disruption of upstream autophagy mediators (e.g. *PPM1E*, *SGK1*, *PRKACA*) displayed profiles like those of autophagy induction (specifically, accumulation of LC3 puncta, reduced p62 intensity, decreased LC3 spot area and increased relative spot intensity)^20,21^. In contrast, disruption of the VPS34 complex core gene *PIK3C3* and its regulatory subunit *PIK3R4*, which are critical for autophagy initiation and phagophore formation, demonstrated a marked increase in p62 intensity, decreased LC3 puncta number, and increased LC3 spot area, fitting the profile of autophagy deficiency (Fig. 2c). Phosphoinositides implicated in autophagosome maturation or autolysosome function (e.g. *FIG4, MTMR14*, *INPP5E*) showed accumulation of bright, smaller LC3 puncta and enlarged LAMP1-positive spots (Fig. 2c). Disruption of *SIRT2*, *SIRT3*, and *SIRT5* gave a profile consistent with previous findings that SIRT2/3 inhibit autophagy under basal conditions via deacetylation of autophagy related proteins^22^. Sirtuins also showed a strong reduction of LAMP1 spot area and intensity (Fig. 2c), suggesting a role in lysosome function.

These results also enabled the prediction of the role of specific genes in the autophagic process via machine learning for the entire screened 1525 gene set. A training gene set was built based on literature reports, and the Support Vector Machine (SVM) learning method was used to predict genes that act similarly to phosphoinositides and sirtuins (Fig. 2e). The trained SVM model had a 70% classification rate of the input training gene sets for phosphoinositides and 75% for sirtuins, with a 5% false discovery rate. A representative list is shown (Fig. 2d) for genes that act similarly to *FIG4* and *MTMR14*, which includes additional correctly predicted genes such as *INPP1*, *MTM1*, *OCRL*, *SACM1L*, *TPTE* and *TPTE2*, all of which have literature support for roles in autophagosome regulation. Moreover, SVM predicted with 90% accuracy genes involved in phospholipid dephosphorylation in the library, 61% for phosphatidylinositol biosynthetic process and 41% for regulation of phosphatidylinositol 3-kinase, while as a negative control non-related histone lysine demethylation was predicted only 5% (Supplemental Table 2). When characterizing predicted sirtuin-like genes in lysosome phenotype, we found two gene categories that were enriched: MAPKKK activity and regulation of gene expression (Fig. 2f). This epigenetic component is further substantiated by GSEA of lysosome phenotypes described below. Ranked gene list based on LAMP1 staining intensity revealed a salient role of epigenetic regulators in modulating lysosome homeostasis (Fig. 2g). In particular, we uncovered a novel bidirectional impact on lysosomes by demethylase (increased LAMP1 intensity) and methyltransferase activity (decreased LAMP1 intensity). Actin binding and cytokine receptor binding activity were also implicated in lysosome modulation (Fig. 2g).

### Discovery of interconnected networks implicating inflammatory NF-κB pathway and LKB1 complex in tau aggregation modulation

Tau aggregation was assessed by quantifying tau aggregate spot area, which was the most sensitive and consistent metric. Disruption of the GSK3A/B genes, which are kinases well studied for their role in tau hyperphosphorylation, disassembled tau aggregates, validating the approach (Fig. 3a). We report for the first time that the *CAB39* gene, which encodes the LKB1 complex component MO25, drastically increased tau aggregation upon CRISPR editing (Fig. 3a). Cytoscape analysis to reveal protein–protein interactions and pathway enrichment among tau aggregation hits showed that KO of all components of the LKB1 complex, *CAB39*, *STK11*, and *STRADA,* significantly increased tau aggregation (Fig. 3b). KO of the LKB1 downstream substrates *MARK1* and *MARK2*, which phosphorylate tau in AD^23,24^, strongly increased tau aggregation. Two other LKB1 substrates, *BRSK1* and *SIK3*, showed a similar phenotype. (Fig. 3b). These results strongly support a role for LKB1 complex in tau aggregation. To test if acute LKB1 perturbation leads to the same result, we used siRNA method to knock down the LKB1 components *CAB39*, *STRADA* and its substrate *MARK2*. Separate knockdowns of around 60% increased tau aggregation by 50% for each of the genes (Fig. 3e). Over-expression of the LKB1 components and *MARK2* using lentiviral ORF constructs gave the opposite phenotype, with significantly decreased tau aggregation (Fig. 3f). Overall, multiple lines of evidence suggest that the LKB1 complex and its substrates modulate tau aggregation in this system.

STRING network analysis shows interactions between genes that increase and decrease tau aggregation bridged by the *FYN* gene, reported to be a downstream mediator of Aβ synaptic toxicity and has been clinically targeted in AD^25^. In our screen, *FYN* disruption also decreased tau aggregation (Fig. 3b). We discovered a large network that reduces tau aggregation and is enriched for the NF-κB signaling pathway. Disruption of the IκB kinase (IKK) complex, which would block NF-κB activation and downstream signaling, significantly reduced tau aggregation. This is supported by the markedly decreased tau aggregation phenotype elicited by loss of all three members of the IKK complex, IKKα (*CHUK*), IKKβ (*IKBKB*) and NEMO (*IKBKG*) in the primary screen (Fig. 3b) and confirmatory experiments (Fig. 3c). Additional NF-κB pathway-associated genes include *ZAP70*, *LYN*, *CSNK2A1* and *CARD11*.

Centering around the IKK complex, and intertwined with the NF-κB pathway, another pathway emerges—cytokine response. CRISPR disruption of chemokines (*CCL2*, *CCL8*), cell surface receptors (*TLR4, TNFRSF1A*, *CD40*, *CD40LG*, *CD14*), inflammatory adhesion molecules (*CD2*, *VCAM1*), and downstream signaling molecules (*IRAK2*, *RIPK1*, *FYN*, *CSNK2B*) all reduced tau aggregation (Fig. 3b,c). This puts the NF-κB finding in an inflammatory context, which by itself is not surprising since NF-κB activation stimulates cytokine production and mediates inflammatory processes. It is however perplexing that such a strong inflammatory involvement in tau aggregation is found in SH-SY5Y cells, a non-immune cell type. We hypothesized that tau aggregation itself may be pro inflammatory. To test this, gene expression profiles of SH-SY5Y cells with or without tau aggregation were compared by RNA-seq. Under normal conditions, SH-SY5Y cells minimally express cytokines, indicated by RPKM values less than 1 (Fig. 3d). However, in tau aggregate-bearing cells, several cytokines increased substantially: *CCL2* (zero to RPKM value of 63), *CCL27*, *IL17D* and *IL32* (zero to RPKM value of 8.4). TNF family receptors also were significantly upregulated: *TNFRSF9* (100-fold increase), *EDA2R* and *FAS*. *IFI44*-interferon induced protein 44-also increased by 100-fold and there was a tenfold increase in the senescence markers IGFBP3 and CDKN1A (Fig. 3d). These results support the hypothesis that tau aggregation induces inflammatory responses, even in SH-SY5Y cells, and that reducing inflammatory signaling decreases tau aggregation in those cells.

### Interplay of tau aggregation with autophagy and lysosome dynamics

Evidence for autophagy and lysosome abnormalities have been found in AD brain and likely impact tau aggregation^26^. PHF seeding to induce tau aggregation resulted in a significant increase in LC3 puncta/spot area, suggesting that aggregation causes stress in the autophagy system, and that interplay of tau aggregation with cellular biological processes can be recapitulated in vitro (Fig. 4a). Treating cells with the autophagy inducers rapamycin and Torin1 had a minor effect on tau aggregation (not shown), but the PI-3K inhibitor wortmannin gave a concentration-dependent increase in tau aggregation area (Fig. 4b). Similarly, CRISPR perturbation of *PIK3CA*, *PIK3CD*, *PIK3CG* and *PIK3C2B* all led to increased tau aggregation, together with a blocked autophagy profile (Fig. 4f). These results suggest that inducing autophagy without increasing autophagic flux is not enough to curb tau aggregation^27^, while inhibition of basal autophagy exacerbates tau aggregation burden.

LAMP1 positive lysosome clusters surrounding tau aggregation spots were frequently observed, and their intensities were inversely related (Fig. 4d). This could indicate lysosome containment of tau aggregates, or exclusion of lysosomes from the dense tau aggregate protein network. However, normal lysosomes appeared as distinct puncta rather than big clusters. To distinguish these possibilities, we performed live imaging experiments to monitor real-time tau aggregation and lysosome dynamics with LysoTracker labeling. PHF treatment resulted in significant increase of lysosome size compared to control prior to visible tau aggregate formation (Fig. 4c,e). Lysosomes continued forming clusters as tau aggregates grew. In some cases, lysosomes formed large clusters and appeared to segment tau aggregates (Fig. 4e). These observations were confirmed with LAMP1-RFP labeled lysosome live imaging. SH-SY5Y cells differentiated into neurons, which minimizes cell movement and allows better tracking of lysosome dynamics in situ, revealed LAMP1-RFP lysosomes trafficking along neurites, coalescing in the cell body to form big clusters in parallel to tau aggregation. The process was highly dynamic within and, unexpectedly, between cells (Supplemental Video 1). These results suggest a strong involvement of lysosomes in tau aggregation. The sirtuin genes, as well as others predicted by machine learning, showed a tau aggregation-high phenotype (Fig. 4f). Example genes include sirtuins (*SIRT2/3/5)*, regulation of MAPKKK activity (*ARAF, LTK*, *MAP3K7CL*, *MAP3K9*), epigenetic regulators of gene expression (*CDK9*, *HDAC4*, *KDM7A*, *MTA3*, *PHF11*, *SETDB2*, *SMARCA2*). Genes involved in glucose metabolism and glycolysis also had a tau aggregation-high phenotype (*PGK1, PKLR*). *PFKFB4* exhibited high tau aggregation together with a sirtuin-like autophagy and lysosome profile (Fig. 4f). Two genes causal for fragile x syndrome, *FMR1* and *FXR2*, displayed a strong lysosome phenotype together with increased tau aggregation upon CRISPR loss of function (Fig. 4f). In summary, tau aggregation in vitro causes stress in autophagy and lysosome dynamics and that modulating genes involved in these processes can affect tau aggregation.

### Mitochondrial morphology indicates cellular bioenergetic preferences and correlates strongly with transcriptional profiles

**Figure 5:**
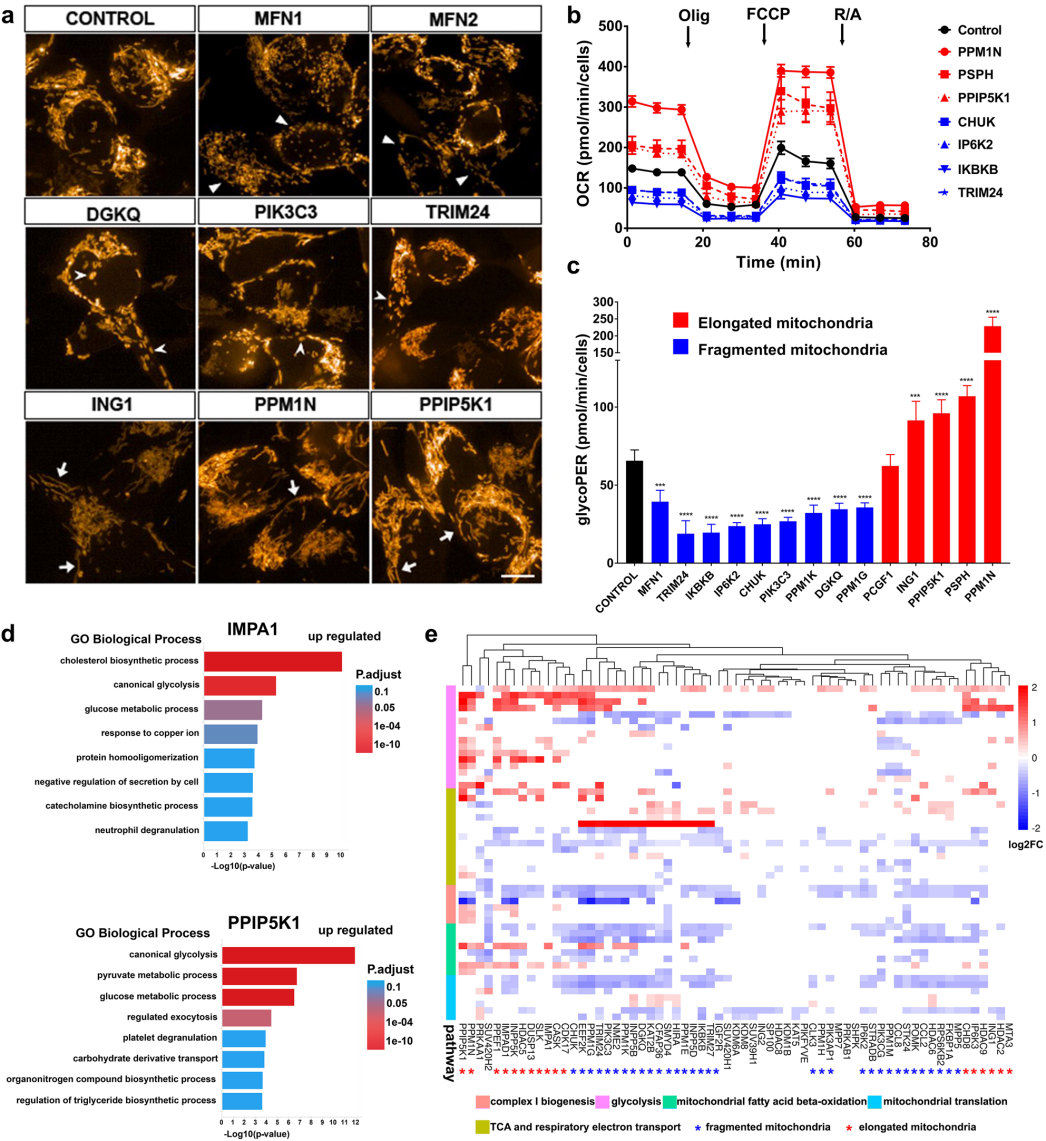
Mitochondrial morphology is correlated with cellular respiration preference and transcription. (**a**) MitoTracker red labeling reveals differences in mitochondrial morphology upon various gene disruptions via CRISPR, broadly categorized as fragmented or elongated mitochondria. Scale bar = 5 μm. Arrowhead points to fragmented mitochondria, open arrows indicate swollen globules and arrows indicate elongated mitochondria. (**b**) Seahorse mito stress test shows markedly different mitochondrial respiration function indicated by cell number normalized OCR, which correlates with mitochondrial morphology, red indicates elongated mitochondria while blue indicates fragmented mitochondria. Olig: oligomycin; R/A: rotenone and antimycin A. (**c**) Seahorse glycolytic rate assay indicates elongated mitochondria is associated with significant upregulation of glycolysis while fragmented mitochondria decreased glycolytic rate based on cell number normalized glycoPER. Error bars indicate standard deviation, n = 6, one-way ANOVA, F (14,60) = 172.8, *p* < 0.0001. Dunnett’s multiple comparisons, ***p-value < 0.001, ****p-value < 0.0001. (**d**) Enrichment analysis of RNA-seq DEGs of cells with elongated mitochondria reveals canonical glycolysis to be the top regulated biological process. (**e**) Hierarchical clustering heatmap of log2 fold change of DEGs involved in mitochondria related processes. Elongated mitochondrial morphology is strongly associated with upregulation of glycolysis genes while fragmented mitochondria have downregulation of genes in involved in TCA, complex I biogenesis and other critical mitochondrial processes.

To inform on cell health and cellular bioenergetics, mitochondrial morphology was monitored by MitoTracker labeling and live imaging. CRISPR disruption of Mitofusin 1 and 2 gave clearly fragmented mitochondria compared to the tubular network observed under basal conditions. *MFN2* disruption also reduced mitochondrial volume compared to *MFN1* and control (Fig. 5a). CRISPR knockout of various genes (e.g. *DGKQ*, *PIK3C3*, *TRIM24*) decreased mitochondrial volume with a different type of fragmentation, swollen globules and rods (Fig. 5a). Others (e.g. *ING1*, *PPM1N*, *PPIP5K1*) led to elongated mitochondria (Fig. 5a).

We used an array of functional assays to investigate whether the observed mitochondrial morphologies reflect function. The Seahorse^28^ Mito Stress Test revealed that cells with elongated mitochondria had higher basal and maximum respiration compared to control measured by oxygen consumption rate (OCR), while cells with fragmented mitochondria showed the opposite (Fig. 5b). The Seahorse Glycolytic Rate assay, which measures proton efflux rate attributed to glycolysis (glycoPER), showed significantly increased glycolysis in cells with elongated mitochondria compared to control, but cells demonstrating mitochondrial fragmentation showed decreased glycolysis (Supplemental Figs. 4 and 5c). The Seahorse ATP Production Rate assay, which measures total ATP production attributed to glycolysis and oxidative phosphorylation (OXPHOS), confirmed that cells with elongated mitochondria upregulated glycolysis (OXPHOS/glycolysis ratios: control: 1.5, elongated mitochondria: 0.5, fragmented mitochondria: > 5, Supplemental Fig. 4). Mitochondrial membrane potential, assessed by TMRE labeling intensity, revealed that cells with fragmented mitochondria have significantly higher membrane potential (2–4 fold, Supplemental Fig. 4), which may explain the increased oxidative phosphorylation capacity. These novel results establish a clear correlation of mitochondrial morphology with cellular bioenergetic preference, particularly that mitochondrial elongation is associated with increased glycolysis.

Cells with altered mitochondrial morphologies were also profiled by RNA-seq and analyzed for transcriptional changes and pathway perturbations. Enrichment analysis of DEGs revealed canonical glycolysis as the top upregulated pathway in cells with elongated mitochondria (e.g. *PPIP5K1*, *IMPA1*), alongside pathways that fit the biological role of these genes (Fig. 5d). Cells with fragmented mitochondria (e.g. *PIK3C3*) downregulated respiratory electron transport chain, complex I assembly, mitochondrial translation and transport (Supplemental Fig. 4d). Similar phenomena were observed across multiple samples, hence a panel of genes critical for the above processes was selected for hierarchical clustering of all samples. CRISPR edited cells with elongated mitochondria clustered with universal upregulation of glycolysis genes (Fig. 5e). For some genes (*ING1*, *HDAC2* and *MTA3*), the edit-induced mitochondria related transcriptomic changes were only limited to upregulated glycolysis genes. Significant down regulation of genes in mitochondrial complex I biogenesis, mitochondrial fatty acid beta-oxidation, mitochondrial translation, the tricarboxylic acid (TCA) cycle and respiratory electron transport were seen in cells with fragmented mitochondria. Cells with CRISPR edits that did not result in an overt mitochondrial morphology phenotype had limited mitochondrial gene network changes (Fig. 5e). Overall, the evidence suggests that mitochondrial morphology is highly correlated with gene expression profiles and that mitochondrial elongation is accompanied by upregulation of glycolysis genes and glycolytic function.

### Gene function inference and fingerprinting based on CRISPR cellular features

Arrayed CRISPR phenotypic screening permits grouping of genes based on similarity of cellular features and inferring novel gene functions and relationships. We chose 33 non-redundant HCI parameters within 6 categories of cellular features quantifying: general cell morphology and cell health, tau, autophagy, mitochondria, Golgi and lysosome. These parameters represent the full spectrum of probed processes and enable identification of communication between pathways. For example, parameters such as tau intensity in aggregate spots vs. LAMP1 positive spots, soluble tau intensity and p62 intensity in LAMP1 spots, might reveal autophagy and lysosome-mediated tau aggregate degradation (Supplemental Table 1).

**Figure 6:**
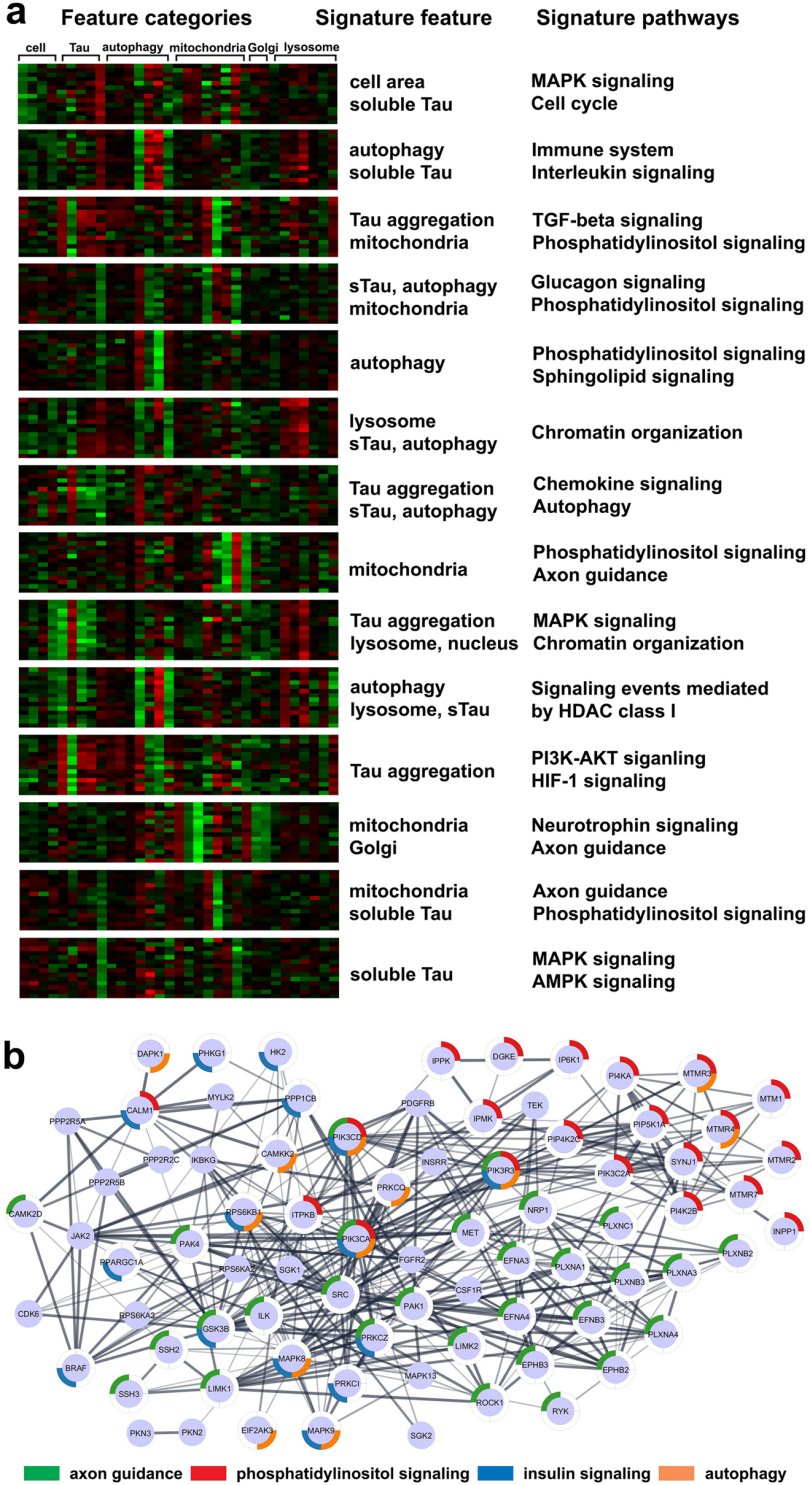
Gene function inference and pathway interactions revealed by signature cellular features. (**a**) Heatmap illustration of k-means clustering of screened genes based on 33 cellular features that capture general cell morphology and health, tau, autophagy, mitochondria, Golgi, and lysosome. The 14 clusters show dominant perturbed cellular processes and are associated with signature enriched pathways. Enriched pathway FDR q-value < 0.05. (**b**) Cytoscape STRING network of genes and pathways that affect mitochondrial morphology. Outer ring highlight network enriched pathway. STRING interaction evidence included experiments and databases.

K-means clustering was used to separate all screened genes into14 groups (Fig. 6a). Each was characterized by dominant cellular features and had specific enriched signature pathways. For example, group 1 was enriched in MAPK signaling (*MAP4K1*, *MAPKAPK2*, *MAPKAPK5*, *MAP3K7*, *MAPK4* and *MAP4K4*), and was associated with bigger cell size and lower soluble tau phenotype (Fig. 6a). A strong mitochondrial morphology phenotype was found in gene sets involved in phosphatidylinositol signaling, axon guidance, insulin signaling and autophagy (Fig. 6a). A detailed STRING interaction network of these gene sets and pathway enrichment is shown (Fig. 6b). The connection of mitochondrial morphology with autophagy echoes the previous characterization, that loss of autophagy-related genes such as *PIK3C3*, *PIK3AP1*, *PIK3CA* resulted in a profound change in mitochondrial morphology, suggesting adaptation and functional metabolic reprogramming. Loss of function of insulin signaling genes (e.g., *PHKG1*, *HK2*, *PRKCZ, PPARGC1A*) also altered mitochondrial morphology. This is consistent with observations that insulin resistance is associated with altered mitochondrial dynamics that favors fission^29^. Genes involved in axon guidance had a strong impact on mitochondrial morphology, including the following gene sets: axon guidance cues ephrins (*EPHB2/3*, *EFNA4*); classic axon guidance molecule semaphorin receptors such as *NRP1* and plexins (*PLXNA1/3/4*, *PLXNB2/3*, *PLXNC1*); growth cone actin polymerization genes (*LIMK1/2*, *SSH2/3*); cytoskeleton dynamics Rho GTPase associated genes (*ROCK1*, *RYK*, *PAK4*); and cell adhesion related genes (*ILK*, *SRC*, *MET*). While it is known that rapid growth cone remodeling during axon outgrowth is energetically costly and requires mitochondrial biogenesis^30^, we report that CRISPR disruption of an array of axon guidance molecules actually alters mitochondrial morphology (Fig. 6b). Our data suggest an intrinsic link between axon guidance biology and mitochondrial dynamics.

**Figure 7:**
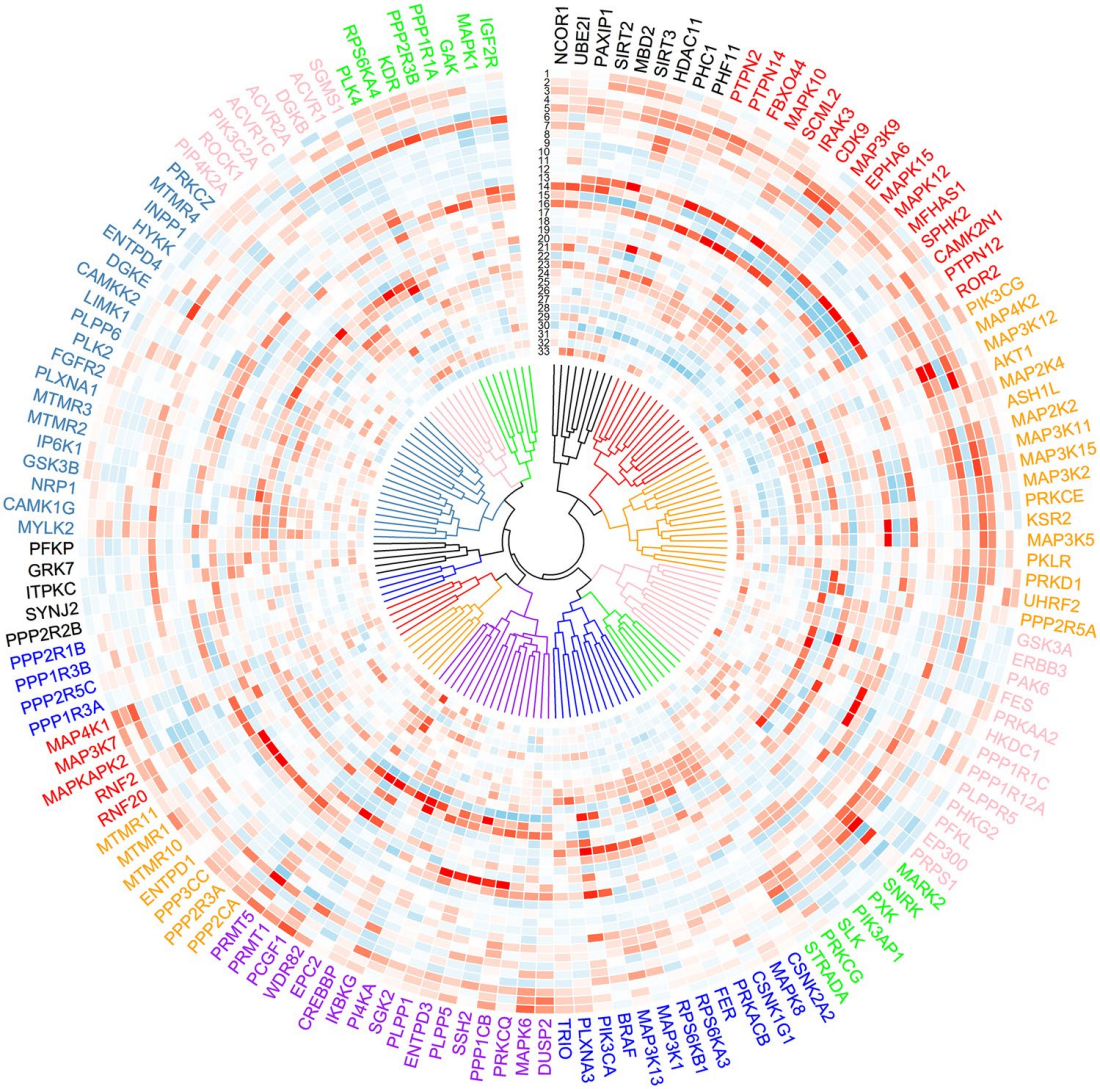
High-resolution finger printing of gene function via hierarchical clustering. Hierarchical clustering of representative genes based on screened 33 cellular features, representing general cell morphology and health, tau, autophagy, mitochondria, Golgi, and lysosome, show similarity of gene functions manifested by probed biological processes. Color coding indicates z score values ranging from − 2 to 2.

Compared to k-means clustering, which extracts dominant cellular features, hierarchical clustering achieves high resolution gene fingerprinting, based on gene similarity indicating related functions in a majority of the probed biological processes (Fig. 7). Hierarchical clustering placed many genes within families adjacent to one another, for example phosphatase regulatory subunits (*PPP1R3A*, *PPP2R5C*, *PPP1R3B*, *PPP2R1B*) and activin receptors (*ACVR1*, *ACVR1C*, *ACVR2A*) Lesser-known genes (e.g., *SLK*, *SNRK*, *PXK*) associate with better characterized genes (*STRADA*, *MARK2*, *PIK3AP1*, *PRKCG*) linked via common tau phenotype (Fig. 7). Detailed examination of this dendrogram will suggest hypotheses for novel gene function by inferences from neighboring genes and respective probed biological processes.

## Discussion

We describe for the first time, a large-scale, arrayed CRISPR screen for genetic modulators of multiple morphological and functional cellular phenotypes associated with AD. We identified novel genes and implied pathways that broaden our understanding of the underlying biology of AD and may be useful as drug targets.

The screen used automation to reduce systematic error, increase throughput, and most importantly, enable parallel quantification of phenotypes from the same batch of CRISPR edited samples. Consistent and high level CRISPR editing was achieved by multiplexing lentiviral gRNAs, which outperformed the best single gRNAs. Similar approaches for improving homology-directed repair (HDR) efficiency have been noted^31^. Systematic gene function profiling is a concept employed by previous studies: RNAi and high content imaging based screens^32,33^, cell painting profiling of small molecules via morphological signatures indicated by multifluorescent probes^34^, and ORF morphological mapping of gene functions by cell painting^35^. This work complements previous studies by choosing a more target specific CRISPR method, shown here to reflect the biological roles of genes, and includes multimodal disease relevant phenotypes. By combining cellular profiling with gene expression profiling, we revealed the complexity of individual gene function and broader pathway involvement. For example, genes linked to axon guidance, phosphatidylinositol signaling, insulin signaling, and autophagy are associated with distinct mitochondrial morphology, and this cellular phenotype is associated with signature mitochondrial gene expression profiles. A systematic analysis pipeline with various bioinformatic tools was developed to extract novel biology from big datasets. For example, machine learning successfully predicted gene functions in discrete stages of autophagy with a multi-parameter SVM linear classifier. These insights provide multiple starting points for further studies.

Numerous novel observations were made. Many autophagy studies focus on mechanisms of stimulated autophagy, which was originally proposed as a stress response^36^. We found phosphatidylinositol signaling to be a major player in basal autophagy, possibly reflecting its role in membrane dynamics crucial for autophagy^37,38^. Mutations in phosphoinositides increase the risk for several neurodegenerative diseases^39^. *FIG4* mutations lead to neurodegenerative diseases such as Charcot-Marie-Tooth disease and Amyotrophic Lateral Sclerosis (ALS). Similarly, *OCRL*, *INPP5B*, *MTM* and *MTMR14* have been linked to various diseases^39^. The fact that all these genes had autophagy alterations in our screen suggests basal autophagy as an underlying cellular mechanism. There is a growing interest in the relationship between mitochondrial morphology and mitochondrial function^40^. We found that elongated mitochondrial morphology is associated with increased glycolysis relative to oxidative phosphorylation and that these functions were associated with an upregulation of glycolysis related gene expression. Previous reports suggested elongation as a mechanism to protect mitochondrial damage during starvation-induced autophagy^41^. We extend this finding with numerous autophagy related gene CRISPR knockouts, demonstrating fragmentation of mitochondria when autophagy is compromised. This suggests a dynamic interaction of biological processes to maintain cellular homeostasis, and that metabolic adaptation and reprograming happens if one process is chronically compromised.

The contribution of autophagy and lysosome biology to the process of tau aggregation was examined in detail. A cellular model of tau pathology similar to ours showed no autophagy impairment during tau aggregation as judged by LC3 and p62 accumulation^15^. We found increased spot area of both LC3 and LAMP1, indicating autophagic and lysosomal stress during tau aggregation, consistent with AD pathology of autophagic vacuoles in dystrophic neurites^42^. Inhibiting basal autophagy with the PI3K inhibitor wortmannin and by disrupting PI3K genes led to increased tau aggregation. This aligns with the consensus that autophagy plays a role in tau aggregation and clearance^27,43^. Intriguingly, a strong link between lysosomes and tau aggregation was found. Lysosomes formed clusters around tau aggregates and modulated tau aggregate morphology. These clusters might come from lysosome coalescence, a mechanism of lysosome enlargement during drug inhibition^44^. Transcription factor EB (TFEB) is a master regulator of lysosome biogenesis^45^. We uncovered a bidirectional modulation of lysosomes by epigenetic regulators. It will be interesting to see if these genes impact lysosomes via TFEB dependent pathways or through new mechanisms. These epigenetic genes also had intriguing effects on tau aggregation. Whether this is related to autophagy-lysosome mediated degradation, lysosome biogenesis, or other mechanisms, requires further study^27,43^.

There is significant evidence that the LKB1 complex modulates tau aggregation. LKB1 is a master kinase regulator with 14 known substrates including AMPK and various tau kinases such as MARK2 and NUAK1^46^. CRISPR disruption of all three components of LKB1 causing increased tau aggregation may reflect the concerted action of tau kinases and autophagy in regulation of aggregation propensity and clearance. We show for the first time, that inhibition of NF-κB, a transcription factor most commonly associated with immune cell activation, can decrease tau aggregation in a neuronal system. Transcriptomic analysis on tau aggregate-bearing cells also shows increased inflammatory signaling, including cytokines, TNF, and interferon related signals. This perhaps identifies a key mechanistic link between inflammation and propensity for tau aggregate formation, as both amyloid beta and proinflammatory cytokines have been described to activate NF-kB in neurons^47^. The neuroinflammation hypothesis of AD is supported by genetic, biomarker, and experimental evidence including recent single cell studies^48^. Most literature suggests that the inflammation signal is due to extracellular Aβ and modulates amyloid progression via microglia^48^. Recently it was reported that knocking out the NLRP3 inflammasome also reduced tau pathology downstream of Aβ through microglia^49^. There is limited literature regarding inflammatory signal response by neurons, despite the presence in neurons of inflammasome components such as NLRP1^50^. Our work suggests that genes traditionally implicated in inflammation can modulate tau aggregation in response to a proinflammatory extracellular environment, and thus may represent a point of intervention. We also reveal potential mechanisms by which the neuronal cell-autonomous inflammatory response that occurs as a result of tau aggregation can trigger the production of chemokines inviting distant and nearby immune cell activation. For example, sequencing shows that tau aggregate-bearing cells produce the chemokine CCL2, which has been described in rodent studies as a key signal to induce macrophage recruitment from the blood into the brain^51^. Together, this work describes specific signaling that modulates propensity for tau aggregation in response to inflammation, as well as how cellular responses to tau aggregation can result in the promotion of neuroinflammation, potentially triggering a feed-forward cascade. Indeed recent literature suggests that perhaps not amyloid itself but instead the inflammatory response to amyloid is key in precipitating dementia^52^. Future experiments in primary neurons^53^ or human iPSC derived neurons^54^ and co-cultured with microglia with reversible techniques such as CRISPRi may better elucidate the mechanisms behind these findings and suggest novel targets for AD therapy.

## Materials and methods

### Cell culture and lentiviral arrayed CRISPR screen

Wildtype SH-SY5Y cells (ATCC, #CRL-2266) were infected with lentivirus encoding CMV-tau (p301L)-EGFP. Cells were maintained in basal medium (DMEM, high glucose, GlutaMAX supplement) with 10% heat-inactivated FBS, 1 × Pen-Strep and 8 μg/mL Blasticidine for selection. Cells were FACS-sorted for optimal EGFP signal and passaged with selection pressure and frozen before passage 18. All cell culture reagents were purchased from Thermo Fisher Scientific.

Same-passage frozen cells were thawed, passaged and plated on 384-well plates (Greiner, #781094) at a density of 7000 cells per well for the screen. The next day, cells were infected with 2.5 μL lentiviral human gRNA library (Thermo Fisher Scientific) with automated liquid handing on a Bravo pipetting workstation (Agilent), supplemented with 8 μg/mL Polybrene (MilliporeSigma) to increase infection efficiency. 16 h later, Cas9 lentivirus was added at a MOI of 1. Three days after Cas9 infection, 1.2 μg/mL puromycin and 1.2 mg/mL G418 were added to the cell culture medium to select for gRNA and Cas9 double-infected cells. Cells were maintained in selection medium with intermittent replenishment for 11 days before splitting onto 384-well imaging plates (CellCarrier-384 Ultra, PerkinElmer).

### High content imaging

High content images were captured on a PerkinElmer Operetta CLS confocal microscope. Exposure time was within the linear range of signal detection. 7–9 fields of images were captured for each well. Three confocal stacks at intervals of 1.5 μM were acquired for all readouts. Live imaging was carried out at 37 °C, 5% CO_2_.

Maximum projections of confocal Z stack images were used for feature extraction and quantification. PerkinElmer Harmony software was used to build algorithms for various readouts. Algorithms were validated by manual inspection of raw images for definitive signal cell events and minimal false positive and false negatives.

### Immunocytochemistry

Culture medium was evacuated from 384 well imaging plates with a BioTek EL406 plate washer. Cells were fixed with cold methanol at − 20 °C for 15 min. Plates were washed once with 1xPBS before primary antibody incubation at 4 °C overnight in staining solution: 1xPBS, 1% BSA (Thermo Fisher Scientific), 0.2% Triton X-100 (Sigma-Aldrich), 5% goat serum (Jackson ImmunoResearch). Secondary staining was performed in PBS with addition of HOECHST 33,342 (Thermo Fisher Scientific). Plates were washed 3 × with PBS between staining steps. Primary antibodies were: mouse anti-p62 antibody (1:1000 dilution, MBL International, #M162-3); rabbit anti-LAMP1 antibody (1:1000, Cell Signaling Technology, #9091S). Secondary antibodies: highly cross-adsorbed secondary antibodies goat anti-mouse IgG (H + L) Alexa Fluor 568/647 and goat anti-rabbit IgG (H + L) Alexa Fluor 647 (Thermo Fisher Scientific).

### Tau aggregation induction and detection

PHF tau seeds were prepared by a heparin-induced recombinant tau (2N4R with P301L mutation) aggregation method^55^. PHF was batch-sonicated and frozen at a stock of 800 nM. SH-SY5Y cells overexpressing tau (P301L)-EGFP were treated with freshly thawed PHF at 15 nM final concentration overnight. PHF treated cells were then exposed to 0.8 mM Leu-Leu methyl ester hydrobromide (LLME, Sigma-Aldrich) for 1 h, and medium was switched to normal culture medium to allow tau seeds to escape from lysosome and form tau aggregation efficiently^56^. 72 h later, cells were fixed with methanol and stained overnight with mouse anti-p62 antibody and rabbit anti-LAMP1 antibody. Secondary antibodies goat anti-mouse Alexa Fluor 568 and goat anti-rabbit Alexa Fluor 647 were used. Plates were then imaged on a PE Operetta with a 40 × water objective at 512 × 512 resolution.

### Autophagy detection and compound treatments

To monitor autophagy, cells were labeled with Premo Autophagy sensor LC3B-RFP (BacMam 2.0) at a MOI of 10 (Thermo Fisher Scientific, #P36236). 48 h later, live imaging was conducted with a 40 × water objective at 512 × 512 resolution for RFP and EGFP channels. Plates were then fixed with methanol and stained with mouse anti-p62 antibody and goat anti-mouse Alexa Fluor 647. Fixed plates were imaged again with the same setting with additional 647 channel for the screen.

BafA1, chloroquine, rapamycin, Torin1, and wortmannin (Tocris Bioscience) were dissolved in DMSO at stock concentrations resulting in 0.1% DMSO final concentrations. Serial dilutions were carried out with DMSO and then with culture medium to keep the DMSO concentration constant.

### SH-SY5Y neuronal differentiation and lysosome tracking

Engineered SH-SY5Y cells were differentiated into neurons as described^57^ with slight modifications. Cells were switched to a low serum medium (normal culture medium with 1% FBS) for three days. 10 μM all-trans retinoic acid was added for four days. Cells were maintained in neuronal medium (Neurobasal medium supplemented with 1 × N2 and B27) containing 10 ng/mL BDNF. Differentiated cells were infected with CellLight Lysosomes-RFP, BacMam 2.0 (Thermo Fisher Scientific, #C10504) at a MOI of 10. The next day, tau aggregation was induced as described above, and the culture plate was live imaged to monitor lysosomes and tau aggregation dynamics over time.

### Organelle labeling and membrane potential assessments

To monitor mitochondria and Golgi, cells were labeled with 75 nM MitoTracker Red CMXRos and 1 μg/mL WGA, Alexa Fluor 647 conjugate (Thermo Fisher Scientific) and Hoechst (1 to 5000) for 1 h. Live imaging was carried out with a 63 × water objective at 512 × 512 resolution for Hoechst, MitoTracker Red, EGFP and Alexa 647 channels. To assess mitochondrial membrane potential, mitochondria were labeled with 10 nM TMRE (tetramethylrhodamine, ethyl ester) for 20 min and cells were live imaged and quantified for TMRE intensity.

### Sanger sequencing and TIDE analysis

DNA was prepared from CRISPR cell lysates using the MagMAX mirVana Total RNA Isolation Kit (Thermo Fisher Scientific) without DNase addition. PCR primers were designed to span gRNA edited regions, with at least 100 bp on either side and final amplicon size between 500 and 850 bp. DNA samples were amplified by PCR. PCR product purification and Sanger sequencing were conducted by Genewiz. Sanger sequencing chromatograms were used for online TIDE analysis as described^58^.

### siRNA and ORF

Silencer Select siRNAs (Thermo Fisher Scientific) were transfected into SH-SY5Y cells at 15 nM using StemFect RNA Transfection kit (Stemgent). PHF was added the next day and tau aggregation was assessed 96 h post siRNA treatment. Cells were infected with lentiviral ORF vectors (Broad Institute) and underwent antibiotic selection for one week prior to tau aggregation induction and assessment. siRNA knockdown efficiency and ORF overexpression level were evaluated by qPCR.

### Mitochondrial Seahorse assays

Real-time quantification of OCR (oxygen consumption rate) and ECAR (extracellular acidification rate) was performed on the Seahorse 96XFe (Agilent) instrument in pH-defined media and normalized to cell counts by imaging and quantification of cell confluency. SH-SY5Y cells reached 90% confluency at the time of assay in normal culture medium. All plates contained Cas9 infected or gRNA control samples. The Seahorse Glycolytic Rate Assay, Mito Stress Test, and Real-Time ATP Rate Assay kits were used according to the manufacturer’s instructions. Each kit enables use of the OCR and ECAR rates following sequential injections of pharmacologic perturbations to quantify the two main energy-production pathways: oxidative phosphorylation and glycolysis. The Glycolytic Rate Assay utilizes a sequential injection of rotenone/antimycin A (0.5 µM), followed by 2-deoxyglucose (50 mM), to quantify glycolysis. The MitoStress Test uses oligomycin (1 µM), FCCP, then rotenone/antimycin A. The Real-Time ATP Rate Assay uses oligomycin, followed by rotenone/antimycin A. The calculations are listed below: glycolytic proton efflux rate (glycoPER) is the rate of protons released into the extracellular media due to glycolysis. GlycoPER is quantified by subtracting the extracellular acidification from mitochondrial CO2 from the total Proton Efflux Rate (PER). glycoPER (pmol H + /min) = PER (pmol H + /min) − mitoPER (pmol H + /min); glycoATP production rate (pmol ATP/min) = glycoPER (pmol H + /min). The mitoATP production rate (pmol ATP/min) is calculated as follows: OCR_ATP_ (pmol O_2_ /min) * 2 (pmol O/pmol O_2_) * P/O (pmol ATP/pmol O), where O is molecular oxygen and the P/O ratio is the number of ATP molecules synthesized per oxygen atom reduced by an electron pair.

### Total RNA isolation, RNA QC, and cDNA preparation

Total RNA was prepared using the MagMAX mirVana Total RNA Isolation Kit (Thermo Fisher Scientific). RNA integrity (RIN) scores and concentration were determined using an Agilent Bioanalyzer with the RNA 6000 Pico or Nano kits. RIN scores ranged from 7.8 to 10, with a mean of 9.2. RNA samples were normalized to 10 ng/µL with water and reverse transcription was performed for all samples in a 20 µL reaction with Superscript IV VILO (Thermo Fisher Scientific) using 16 µL of each RNA preparation. The cDNA yield for each reaction was determined using Quant-iT OliGreen reagent (Thermo Fisher Scientific). Samples with a cDNA concentration greater than 5 ng/µL were normalized to that value by addition of water.

### Quantitative PCR (qPCR) and data analysis

Samples were subjected to qPCR analysis using the Juno/Biomark HD high throughput platform (Fluidigm). cDNA was preamplified for 15 PCR cycles in a multiplex fashion using the appropriate Taqman assay panel. Following tenfold dilution with water, the preamplified samples were prepared for loading onto the integrated fluidic circuit according to the manufacturer’s protocol. Data analysis was performed using the GenEx Professional software package, version 6, (MultiD Analyses AB). Starting with the raw Ct values, the Normfinder feature of the software was used to identify the most robust normalization scheme, which was used to convert raw Ct values into delta Ct values. Differential gene expression values for each sample were calculated relative to the average expression level of the reference group using the 2^delta.

### RNA-seq analysis

Library preparation for RNA-seq analysis was performed as described^59^ starting with 28 ng of total RNA. Samples used for sequencing had an average RIN of 9.8. Sequencing was performed using a NextSeq550 (Illumina). Sequencing analysis was conducted by polyA-trimming off the reads before alignment. STAR 2.6.0a was used to align the reads to the hg19 reference. FeatureCounts was used to map sequencing reads to genomic features. The number of transcripts per kilobase million (TPM) was used to evaluate gene expression level. We used the R package “DESeq2” to calculate differential gene expression comparing the target phenotypes with control group. The log ratio of genes (log2fold change) was used to compare gene expression levels.

### Bioinformatics analysis

We used heatmaps and hierarchical clustering to find the structures in our gene expression trends and to partition genes into clusters. The heatmap was generated by the R package “pheatmap”. The clustering distance is calculated by the Euclidean method. The transcript expression value is presented by the log ratio of a gene’s log2fold change.

We use principle component analysis (PCA) as the dimensional reduction technique to study the phenotypic patterns with the selected genes TPM (transcripts per kilobase million). PCA is performed by the R package “prcomp”.

The R package “mclust” for model-based clustering was used to cluster cellular features extracted from HCI. The optimal number of groups was used for k-means calculation (nstart = 25, algorithm = "Hartigan-Wong"). The R package “hclust” with method “complete” was used to generate a circular dendrogram for cellular feature hierarchical clustering.

Cellular feature ranked z score enrichment analysis was conducted by GSEA (Broad Institute version 3.0) using weighted enrichment statistic by 1000 permutations, with FDR = 0.05 as the threshold. GO Biological Process, GO Molecular Function, and KEGG pathways^60^ were utilized.

Ranked DEGs with adjusted p value cutoff of 0.05 were used for pathway enrichment analysis via Enrichr platform^61^. KEGG^60^, BioPlanet, and Reactome pathways were utilized. Protein–protein interaction network and enrichment analyses were conducted by Cytoscape with STRING app^62^. STRING interactions were only limited to experiments and databases.

Support vector machine was used as a machine learning method by importing the R package “e1071”. The training data was curated by annotating 67 genes with their significant functions from various literature. The training strategy was to highlight the targeted category and temporarily label other categories as the ’rest’ ("one-against-the-rest"). The binary classification was based on linear kernel (type = C-classification, cost = 10, scale = False). The trained model was used to predict screened genes without prior notification for their functions. All hyperparameters of these genes were PCA transformed and plotted in 3D space by their first three principal components (PC1 var = 51.3%, PC2 = 17.4%, PC3 = 12.1%).

## Supplementary Information

Supplementary Information.

Supplementary Video.
