# Supplementary material for: Arrayed CRISPR reveals genetic regulators of tau aggregation, autophagy and mitochondria in Alzheimer’s disease model
Source: Sci Rep. 2021 Feb 3;11:2879. doi: 10.1038/s41598-021-82658-7 (PMC7859211; doi:10.1038/s41598-021-82658-7)
Supplement: Supplementary file 1 — Supplementary Information. [file 41598_2021_82658_MOESM1_ESM.pdf]

Supplemental information

Arrayed CRISPR reveals genetic regulators of tau aggregation, autophagy and mitochondria in Alzheimer's disease model

Lishu Duan[1*], Mufeng Hu[2], Joseph A. Tamm[1], Yelena Y. Grinberg[1], Fang Shen[1], Yating Chai[2], Hualin Xi[3], Lauren Gibilisco[2], Brinda Ravikumar[1], Vivek Gautam[1], Eric Karran[1], Matthew Townsend[1], Robert V. Talanian[1]

[1]AbbVie, Cambridge Research Center, 200 Sidney Street Cambridge, Massachusetts, USA 02139.

[2]AbbVie Inc., 1 North Waukegan Rd., North Chicago, IL 60064.

[3]Former AbbVie employee.

*Lishu.duan@abbvie.com

Supplemental Table 1: HCI features

Feature category	Feature order	Feature name
Cell	1	Cell area
Cell	2	Nucleus area
Cell	3	Nucleus intensity
Cell	4	Nucleus number
Tau	5	Tau aggregate spot area
Tau	6	Tau aggregate spot to region intensity
Tau	7	Tau intensity in aggregate spots
Tau	8	Tau intensity in LAMP1 spots
Tau	9	Soluble tau intensity
Autophagy	10	P62 intensity
Autophagy	11	P62 intensity in LAMP1 spot
Autophagy	12	P62 spot area over cell area
Autophagy	13	LC3 puncta number low
Autophagy	14	LC3 puncta number high
Autophagy	15	LC3 relative spot intensity
Autophagy	16	LC3 spot area
Mitochondria	17	Mitochondria area
Mitochondria	18	Mitochondria width to length ratio
Mitochondria	19	Mitochondria roundness
Mitochondria	20	Percent elongated mitochondria
Mitochondria	21	Percent fragmented mitochondria
Mitochondria	22	Percent high volume mitochondria
Mitochondria	23	Percent low volume mitochondria
Mitochondria	24	Number of mitochondria per cell area
Golgi	25	WGA relative spot intensity
Golgi	26	WGA spot per area of cell
Lysosome	27	Cytoplasmic LAMP1 intensity
Lysosome	28	LAMP1 spot corrected intensity
Lysosome	29	LAMP1 spot area
Lysosome	30	LAMP1 total spot area
Lysosome	31	LAMP1 spot area over cell area
Lysosome	32	Nuclear LAMP1 spot event
Lysosome	33	Big LAMP1 spot event

Supplemental Table 2: Machine learning prediction accuracy

GO Biological Process	phosphatidylinositol biosynthetic process	SVM results	phospholipid dephosphorylation	SVM results	regulation of phosphatidylinositol 3-kinase signaling	SVM results	phosphatidylinositol-mediated signaling	SVM results	histone lysine demethylation	SVM results
Percentage of predicted genes	61%		90%		44%		38%		5%	
	MTMR12	predicted	MTMR1	predicted	FLT3	predicted	CSF1R	predicted	KDM1A	predicted
	MTMR14	predicted	MTMR2	predicted	EGFR	predicted	PIK3R2	predicted	KDM6B	
	INPPL1	predicted	MTMR3	predicted	ERBB3	predicted	ERBB3	predicted	KDM5A	
	IMPA1	predicted	PLPPR1	predicted	KDR	predicted	KCNH1	predicted	KDM5B	
	PIP4K2B	predicted	PLPPR2	predicted	PIP4K2B	predicted	PDGFRA	predicted	KDM4A	
	SBF1	predicted	PLPPR3	predicted	SOX9	predicted	PIK3CA	predicted	KDM4B	
	TPTE2	predicted	PLPPR4	predicted	PPP1R16B	predicted	TYRO3	predicted	KDM3A	
	SACM1L	predicted	SACM1L	predicted	PDGFRA	predicted	PI4KA	predicted	KDM5C	
	OCRL	predicted	PLPPR5	predicted	ENTPD5	predicted	PIK3C3	predicted	KDM2A	
	PIK3CA	predicted	MTM1	predicted	PPP2R5B	predicted	FGFR1	predicted	KDM4C	
	PIK3C3	predicted	INPP5B	predicted	PPP2R5C	predicted	LTK		KDM5D	
	PI4K2A	predicted	PLPP6	predicted	PTPN13	predicted	PIK3CD		KDM3B	
	MTMR1	predicted	PLPP5	predicted	PTPN6	predicted	PIK3C2G		KDM4D	

	MTMR2	pred icte d	PLPP4	pre dict ed	FGFR1	predi cted	PIK3CB		KDM1B	
	MTMR3	pred icte d	INPP5K	pre dict ed	FLT1		PIK3C2A		KDM4E	
	MTMR8	pred icte d	PLPP3	pre dict ed	CCL5		PIK3CG		KDM8	
	PIK3R4	pred icte d	PLPP2	pre dict ed	PIP4K2A		PIK3R6		JMJD1C	
	MTMR9	pred icte d	PLPP1	pre dict ed	PIP5K1A		PIK3C2B		HR	
	PIK3R3	pred icte d	MTMR6		MAPK1		IGF1R		PHF8	
	PIK3R2	pred icte d	SYNJ1		PIP5K1B		ERBB2		KDM7A	
	MTMR4	pred icte d			PIP4K2C		AKT1			
	MTMR7	pred icte d			PIP5K1C		PDGFRB			
	MTM1	pred icte d			JAK2		NTRK1			
	INPP5D	pred icte d			PDGFRB		SIRT2			
	INPP5E	pred icte d			NTRK2		RPS6KB1			
	INPP5J	pred icte d			TGFB2		PI4KB			
	INPP5K	pred icte d			PTK2					
	PTPN13	pred icte d			FGR					
	FIG4	pred icte d			KIT					
	PI4KA	pred icte d			PLXNB1					

	PI4K2B				TEK					
	PIK3CD				PIK3AP1					
	PIK3C2G									
	PIK3CB									
	PIK3C2A									
	PIK3CG									
	PIK3C2B									
	PIP4K2A									
	PIP4K2C									
	MTMR6									
	PIK3R6									
	PIP5K1A									
	PIP5K1B									
	PIP5K1C									
	SYNJ2									
	BMX									
	PIKFYVE									
	SYNJ1									
	PI4KB									

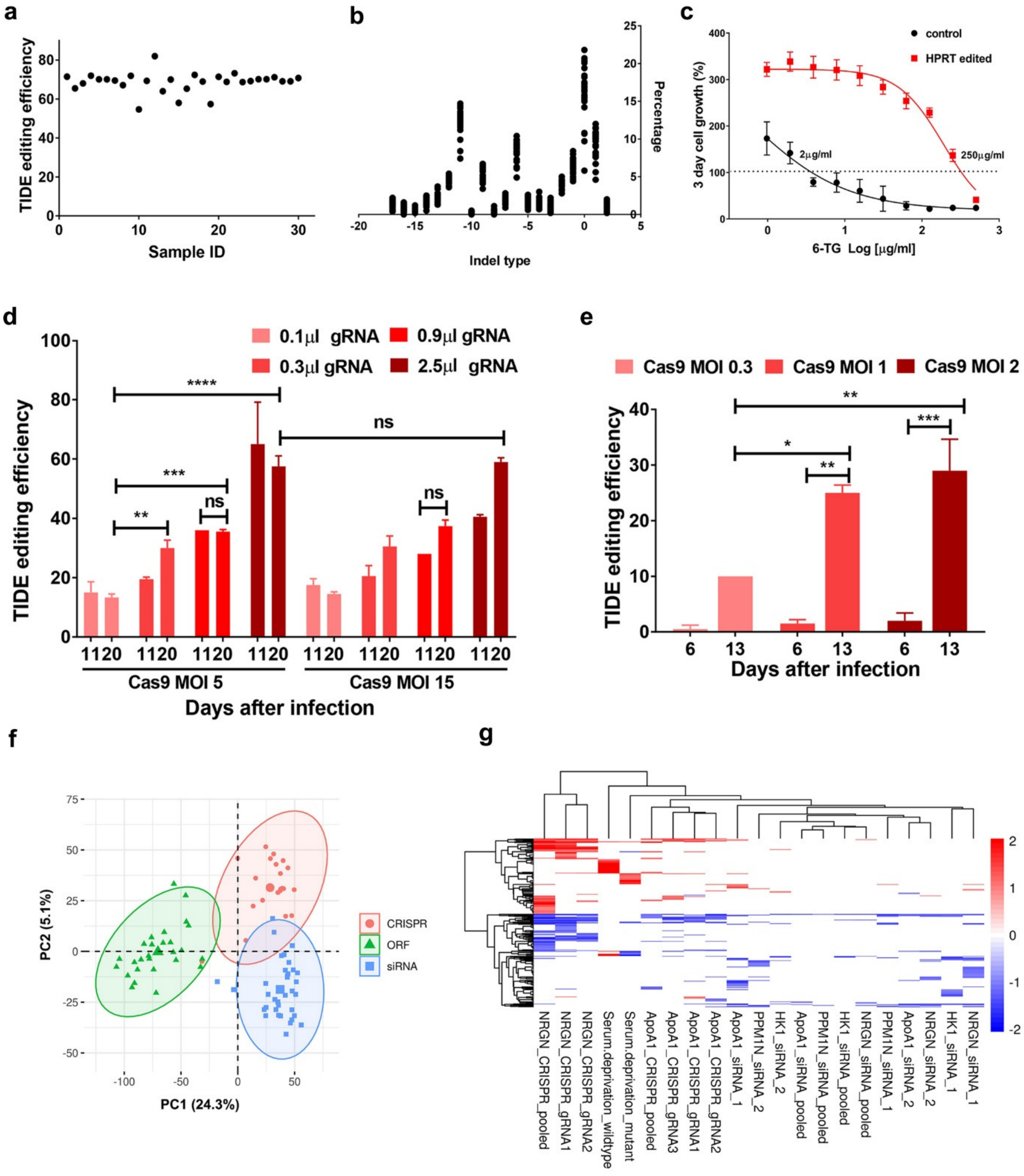

Supplemental Fig. 1: Development of consistent, efficient, specific and translatable arrayed CRISPR method.
a, Singe gRNA against *HPRT* gene editing efficiency is reproducible across 30 samples, mean editing efficiency is 69% with 5% SD, determined by Sanger sequencing and TIDE analysis. b, TIDE analysis of *HPRT* single gRNA reveals guide-specific DNA editing pattern. c, 69% *HPRT* DNA editing efficiency translates functionally into 6-TG resistance, with 350-fold right shift in IC_{50} n=6. d-e, Optimization of lentiviral gRNA volume, Cas9 MOI and editing timeline using gRNA against *ATG7* (d, n=3) and *DLG2* (e, n=2). Two-way ANOVA with SIDAK'S multiple comparisons was applied. f, ORF elicits drastically different gene expression profiles from CRISPR and siRNA method. Evidenced by PCA analysis of gene counts via RNA-seq after various manipulations, n=3. g, Hierarchical clustering of log2 fold change of DEGs show agreement of different gRNAs towards the same gene for CRISPR but not siRNA method, n=3. All error bars indicate standard deviation, * p-value < 0.5, ** p-value <0,01, *** p-value < 0.001, **** p-value < 0.0001.

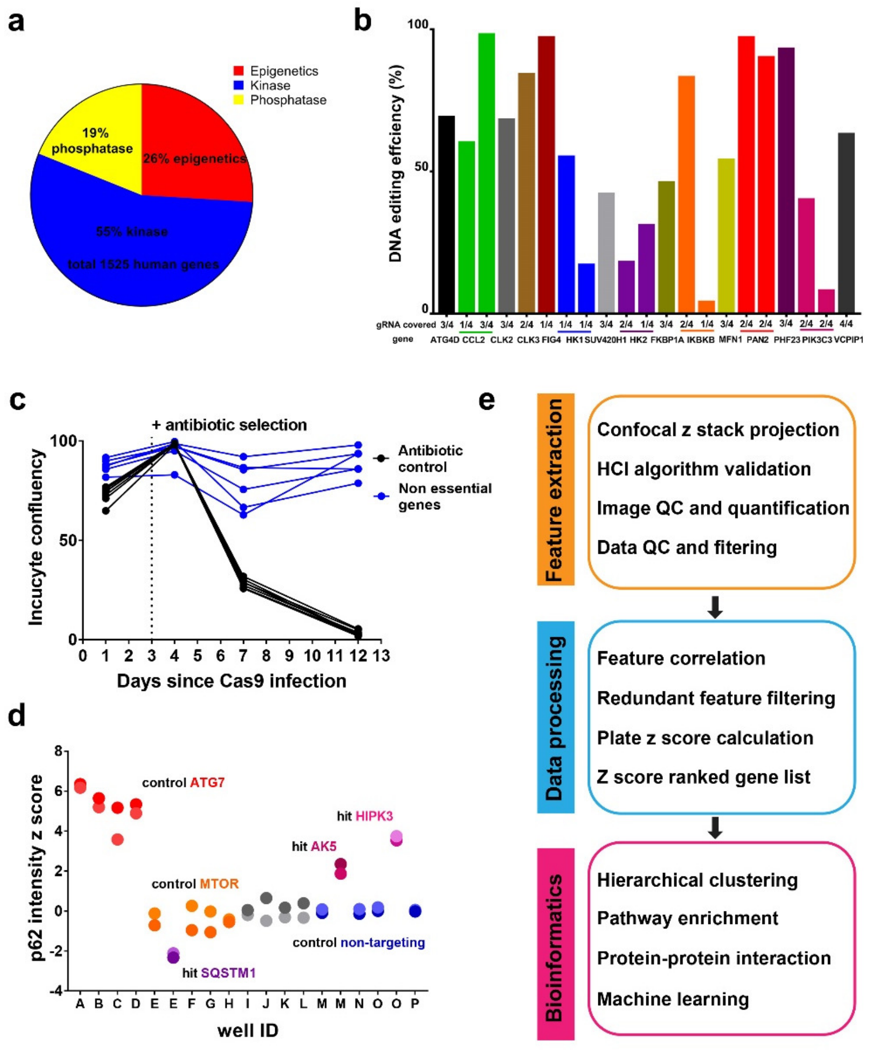

Supplemental Fig.2: Arrayed CRISPR screen performance and data analysis pipeline.
a, Arrayed CRISPR screen library makeup. b, Sampling after CRISPR screen reveal high editing efficiency across the board determined by Sanger sequencing and TIDE analysis. gRNA covered indicates number of gRNAs covered by the PCR fragment used for Sanger sequencing. Each gene had a total gRNA number of 4. c-d, Control performances demonstrating elimination of non-viral infected cells with antibiotic control (c) and expected phenotypes for autophagy controls *ATG7* and *MTOR* (d), a few hits are also illustrated. e, Schematics of image processing and data analysis pipeline.

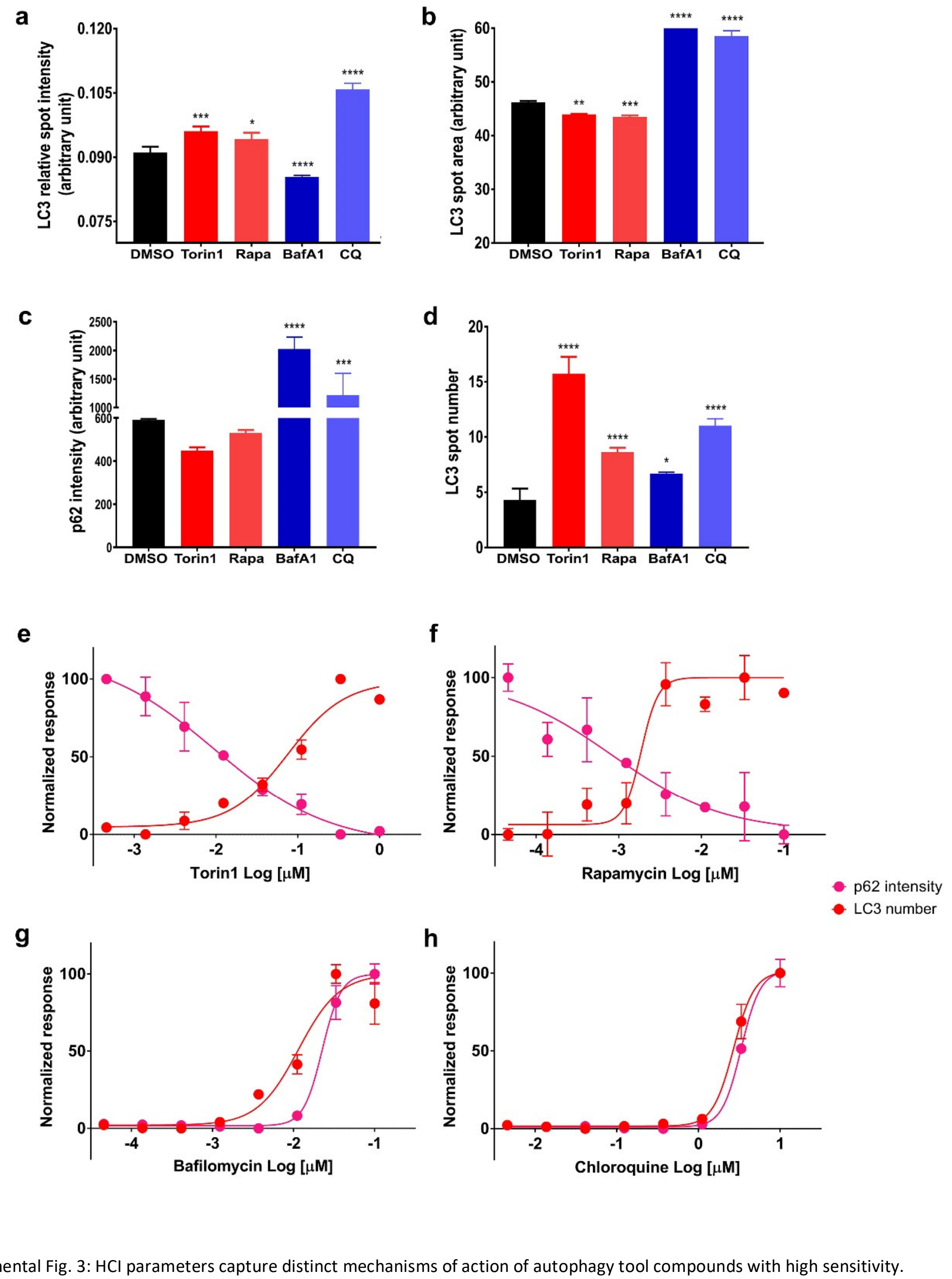

Supplemental Fig. 3: HCI parameters capture distinct mechanisms of action of autophagy tool compounds with high sensitivity.
a-d, Various HCI parameters related to autophagy markers LC3 and p62 capture differences of mechanism of action of autophagy tool compounds. Rapa: rapamycin. BafA1: bafilomycin A1. CQ: chloroquine. N=4, one-way ANOVA with Tukey's multiple comparisons. e-f, mTOR inhibitors Torin1 and rapamycin concentration dependently increased LC3 puncta number while decreasing p62 intensity. g-f, Autolysosome inhibitors bafilomycin and chloroquine concentration dependently increased LC3 puncta number and p62 intensity. All error bars indicate standard deviation, * p-value < 0.5, ** p-value <0,01, *** p-value < 0.001, **** p-value < 0.0001.

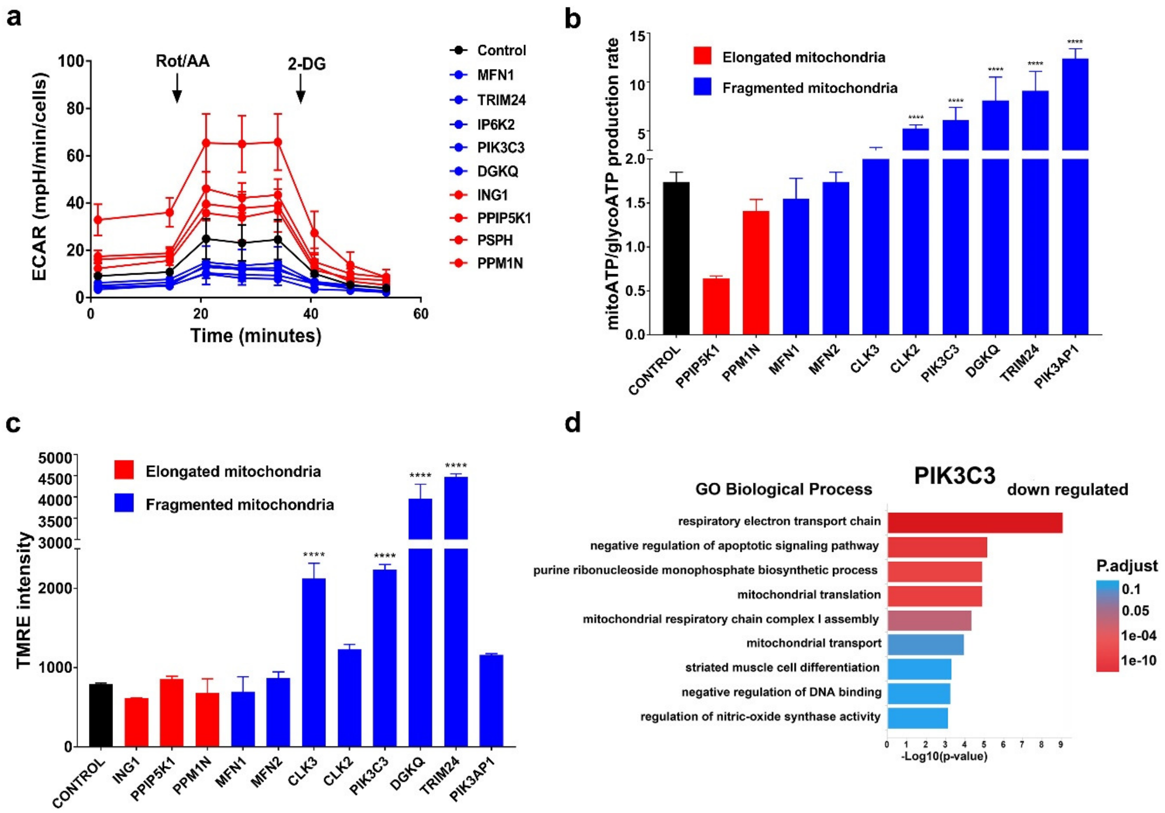

Supplemental Fig. 4: Mitochondrial morphology is correlated with cellular respiration preference and transcription.
a, Seahorse glycolytic rate assay shows higher glycolytic rate in cells with elongated mitochondria evidence by increased cell number normalized ECAR. b, Seahorse real-time ATP rate assay quantified the ratio of ATP generated via glycolysis versus mitochondrial respiration. Elongated mitochondria show shift of cellular respiration towards glycolysis while fragmented mitochondria relied more on mitochondrial respiration. c, TMRE results indicate significant increase of mitochondrial membrane potential with fragmented morphology. d, Enrichment analysis of DEGs upon *PIK3C3* CRISPR knockouts which resulted in fragmented mitochondria, showed down regulated gene sets including TCA, mitochondrial biogenesis, translation and transport. All error bars indicate standard deviation, n=6, one-way ANOVA with Dunnett's's multiple comparisons, **** p-value < 0.0001.

Supplemental Video 1: Lysosome coalesce during tau aggregation process. Live imaging of LAMP1-RFP labeled lysosomes coalescing from peripheral regions into cell body regions as well as between cells in cells treated for tau aggregation.

Note: video file provided separately
